# Pyroptosis and Its Role in Autoimmune Disease: A Potential Therapeutic Target

**DOI:** 10.3389/fimmu.2022.841732

**Published:** 2022-05-25

**Authors:** Ruixuan You, Xinglan He, Zhuotong Zeng, Yi Zhan, Yangfan Xiao, Rong Xiao

**Affiliations:** ^1^ Department of Dermatology, The Second Xiangya Hospital of Central South University, Changsha, China; ^2^ Hunan Key Laboratory of Medical Epigenetics, The Second Xiangya Hospital of Central South University, Changsha, China; ^3^ Clinical Nursing Teaching and Research Section, The Second Xiangya Hospital of Central South University, Changsha, China; ^4^ Department of Anesthesiology, The Second Xiangya Hospital of Central South University, Changsha, China

**Keywords:** autoimmune diseases, pyroptosis, inflammasome, caspases, gasdermin

## Abstract

Autoimmune diseases are a group of heterogeneous diseases with diverse clinical manifestations that can be divided into systemic and organ-specific. The common etiology of autoimmune diseases is the destruction of immune tolerance and the production of autoantibodies, which attack specific tissues and/or organs in the body. The pathogenesis of autoimmune diseases is complicated, and genetic, environmental, infectious, and even psychological factors work together to cause aberrant innate and adaptive immune responses. Although the exact mechanisms are unclear, recently, excessive exacerbation of pyroptosis, as a bond between innate and adaptive immunity, has been proven to play a crucial role in the development of autoimmune disease. Pyroptosis is characterized by pore formation on cell membranes, as well as cell rupture and the excretion of intracellular contents and pro-inflammatory cytokines, such as IL-1β and IL-18. This overactive inflammatory programmed cell death disrupts immune system homeostasis and promotes autoimmunity. This review examines the molecular structure of classical inflammasomes, including NLRP3, AIM2, and P2X7-NLRP3, as the switches of pyroptosis, and their molecular regulation mechanisms. The sophisticated pyroptosis pathways, including the canonical caspase-1-mediated pathway, the noncanonical caspase-4/5/11-mediated pathway, the emerging caspase-3-mediated pathway, and the caspase-independent pathway, are also described. We highlight the recent advances in pyroptosis in autoimmune diseases, such as systemic lupus erythematosus, rheumatoid arthritis, inflammatory bowel disease, Sjögren’s syndrome and dermatomyositis, and attempt to identify its potential advantages as a therapeutic target or prognostic marker in these diseases.

## Introduction

In 1992, pyroptosis was originally defined as macrophage lysis following *Shigella flexneri* infection, although researchers mistakenly regarded it as apoptosis at the time (1). It was not until 2001 that Cookson and Brennan first proposed the term “pyroptosis” to define this process of cell death. Pyro, originating from Greek roots, means fire or fever, which is used to highlight the features of inflammation. Ptosis, deriving from Greek roots, means to “fall off,” is adopted as the common suffix root in cell death (2). As a form of inflammatory programmed cell death, the primary feature of pyroptosis is membrane pore formation, which is dependent on the N-terminal domains of the gasdermin protein family. These domains are often (but not always) cleaved by the activated caspase family, leading to cell swelling, final rupture, and the outflow of IL-1β, IL-18, and cytoplasmic contents (3).

In innate immunity, moderate pyroptosis can eliminate the replication niche of intracellular pathogens, making them vulnerable to killing by innate immune cells and protecting the remaining cells from microbial invasion (4). Nevertheless, emerging evidence demonstrates that the aberrant activation of pyroptosis may initiate autoimmune disease. Pyroptosis is not only a silent type of cell death similar to apoptosis, resulting in the partial loss of the structure and function of tissues or organs, but also leads to the release of abundant inflammatory media at the end of cell life. This prolonged release of inflammatory factors triggers an overactive immune system and leads to the continuation and progression of autoimmune diseases (5). Specifically, the uncontrolled release of pro-inflammatory cytokines assists differentiated mature T cells in inducing adaptive immunity. The subsequent dysregulation of adaptive immunity leads to autoimmune system dysfunction and loss of tolerance to normal tissues and organs. Under these conditions, autoantibodies and/or autoreactive T cells will mistakenly attack the body, causing autoimmune diseases (6–8).

Thus, in this review, we comprehensively elaborate on the pathophysiological mechanism and molecular signal pathways of pyroptosis and its role in the pathogenesis of autoimmune diseases.

## Pivotal Inflammasomes in Pyroptosis

As an immune signaling multi-protein complex, the canonical inflammasome is assembled by a specific sensor, adaptor protein apoptosis-associated speck-like protein containing a CARD (ASC), and effector pro-caspase-1 (9). Thus far, canonical inflammasomes that enable the induction of pyroptosis include NLRP1, NLRP3, NLRC4, AIM2, and Pyrin (10). However, given that NLRP3, AIM2, and P2X7-NLRP3 are the most thoroughly studied in terms of pyroptosis in the context of autoimmune disease, we mainly introduce the biological characteristics of these inflammasomes and discuss their activation and modification patterns.

### NLRP3 Inflammasome

As a representative of the nucleotide-binding leucine-rich repeat proteins (NLRs) family, the NLRP3 protein mainly contains the following three components: a C-terminal leucine-rich repeat (LRR) domain; a central adenosine triphosphatase (ATPase) domain known as NACHT; and N-terminal pyrin domain (PYD) (11). The NLRP3 family also contains a caspase activation and recruitment domain (CARD) at the C-terminus and a PYD at the N-terminus, which assemble into ASC (12). Following detection of pathogens and endogenous danger signals by the LRR, the oligomeric NLRP3 inflammasome gathers together through its NACHT domains and recruits ASC through PYD-PYD interactions to nucleate PYD filaments of ASC. Finally, the adaptor protein ASC attracts pro-caspase-1 *via* CARD-CARD interactions, inducing the self-cleavage of caspase-1 (13).

The activation of the NLRP3 inflammasome is a two-step signal model. The first signal (priming) is provided by Toll-like receptor (TLR) and cytokine receptors, such as leukin-1 receptor (IL-1R) and tumor necrosis factor receptor (TNFR) (14). Following identification of microbes or inflammatory cytokines by NF-κB-activating receptors, NF-κB is immediately translocated to the nucleus with the assistance of FADD and caspase-8, which raises the content of NLRP3 and pro-IL-1β by boosting their gene transcription and translation (14, 15). The second signal (activation) is triggered by extensive stimuli, including pore-forming toxins, ATP, and different particulates (16, 17). Owing to the second step, the NLRP3 inflammasome completes assembly and activates caspase-1, which processes pro-IL-1β and pro-IL-18 into their mature forms at the microtubule-organizing center (MTOC) distributed in the perinuclear and punctate regions (11, 18). It has been suggested that NLRP3 inflammasome activation is mediated by intricate cellular signaling events, including potassium efflux, calcium overload, reactive oxygen species (ROS) generation, mitochondrial dysfunction and lysosomal rupture (19–23). Yet, the specific mechanism of ion flux changes or organelle dysfunction in the activation of the NLRP3 inflammasome remains controversial.

Additionally, NIMA-related kinase 7 (NEK7) has recently been authenticated an essential activator of the NLRP3 inflammasome (24, 25). Initially, histone deacetylase 6 (HDAC6) may carry the NLRP3 inflammasome to the MTOC, where NEK7 is located, with the aid of microtubule transport (18). Subsequently, Sharif et al. found that the curved LRR and globular NACHT domains together made up the earring-shaped NLRP3. The former interacts with the first half of the NEK7 C-lobe, while the latter interacts with the second half of the NEK7 C-lobe *via* its NBD and helical domain 2 (HD2) (26), thus unveiling that even though the NLRP3-NEK7 complex alone is insufficient to support NLRP3 inflammasome activation, NEK7 can be responsible for signal transduction generated by the above stimuli during NLRP3 activation.

Remarkably, the post-translational modification (PTM) of the NLRP3 inflammasome is also an indispensable step in regulating its activity. NLRP3 deubiquitination and ASC ubiquitination or phosphorylation are expected to promote activation, while NLRP3 phosphorylation has dual-directional effects, depending on when and where this modification occurs (27–31). There is a prevailing notion that that high expression of NLRP3 inflammasome is observed in patients with autoimmune diseases; thus, the NLRP3/IL-1 axis is highly susceptible to initiate an overreaction of the immune system.

### AIM2 Inflammasome

AIM2 (absent in melanoma 2), a member of the pyrin and HIN domain-containing (PYHIN) protein family, is composed of a C-terminal HIN-200 domain and an N-terminal PYD. AIM2 senses cytoplasmic DNA *via* its HIN-200 domain, while the PYD combines with adaptor protein ASC whose CARD can summon and activate pro-caspase-1 (32, 33).

As a cytoplasmic DNA sensor, AIM2 has been proven to respond to DNA from various sources, including bacterial DNA, such as *Francisella tularensis, Porphyromonas gingivalisas, Legionella pneumophila, Staphylococcus aureus, Brucella abortus*, and *Chlamydia muridarum* (34); viral DNA, such as human papillomavirus, and enterovirus 71 (EV71) (35, 36); influenza virus-induced oxidized mitochondrial DNA (mtDNA) (37); ionizing radiation-induced DNA (38); and self-DNA released through exosomes (39). In addition to DNA, AIM2 can monitor the invasion of fungi, such as *Aspergillus fumigatus*, protozoans, such as *Plasmodium berghei*, and possibly SARS-CoV-2 (the causative virus of COVID-19) (40–42).

DNA that enters the cytoplasm will have difficulty making direct contact with the AIM2 inflammasome directly given that it is always encapsulated by cell membrane to avoid immune attack. Fortunately, Type I interferon (type I IFN) assumes responsibility for puncturing this protective film and exposing bacterial DNA. Taking *Francisella novicida* as an example, the nucleotidyl transferase, cGAS, induces the expression of type I IFN through the STING-TBK1-IRF3 pathway after detecting foreign DNA (43). Next, the type I IFN signaling formed by the combination of type I IFN and type I IFN receptor (IFNAR) up-regulates the expression of interferon regulatory factor 1 (IRF1) (44). The expression of IRGB10 and guanylate binding proteins (GBPs) especially GBP2 and GBP5, are up-regulated in response to IRF1 induction (45, 46). These interferon-inducible proteins immediately destroy the bacterial membrane of *F. novicida*, such that its DNA can enter the cytoplasm and bind to the AIM2 inflammasome (47). Subsequently, the negatively charged dsDNA sugar-phosphate backbone and the positively charged HIN domain residues rely on electrostatic attraction rather than a DNA sequence to bind (48). Yet, the dsDNA length determines the assembly dynamics of the AIM2 inflammasome. Biochemical cellular studies have illustrated that the threshold length of dsDNA that can provoke AIM2 inflammasome is 80 bp, while 200 bp of dsDNA may achieve the peak. A stepwise-amplified signal, accelerating the formation of AIM2 and ASC filaments, will be generated from AIM2 to ASC as soon as the dsDNA length reaches the conditions that trigger inflammasome assembly (49).

There remain many controversies regarding how the binding of DNA to AIM2^HIN^ leads AIM2^PYD^ to recruit downstream ASC. Jin et al. initially proposed that with the absence of cytoplasmic DNA, the PYD and HIN domains of AIM2 preferred to make up an autoinhibited intramolecular complex; once the HIN domain met dsDNA, the PYD would be replaced and removed from the complex, thereby allowing it to interact with downstream ASC (48). This hypothesis was later challenged by Sohn et al., who demonstrated that the role of AIM2^PYD^ was not autoinhibition, but to oligomerize and impel filament assembly, thus constructing the structural template for downstream ASC^PYD^ polymerization. This novel discovery may be mainly attributed to the fact that ASC^PYD^ filaments have a helical architecture consistent with AIM2^PYD^ filaments (50), which is a prerequisite for the unidirectional recognition between AIM2^PYD^ and ASC^PYD^, permitting the top of the AIM2^PYD^ filament to make contact with only the bottom of the ASC^PYD^ filament (51).

Similarly, due to the plentiful and continuous self-DNA deposition in patients with autoimmune disease, there is a potential threat of AIM2 over-activation. Therefore, it is important for the AIM2 inflammasome to conduct the PTM with the intention of regulating activity. T. Liu et al. reported that tripartite motif 11 (TRIM11) binds to AIM2 through its PS domain and performs polyubiquitination at K458, which could push AIM2 to the autophagic cargo receptor p62 for autophagy-dependent degradation (52). In contrast, HUWE1, originated from the HECT E3 ubiquitin ligase family, was recently discovered to mediate K27-linked polyubiquitination at the lysine residues of the AIM2^PYD^ domain, where it promotes the assembly and activation of the AIM2 inflammasome (53). As ubiquitination is a reversible process, the regulation of deubiquitinase activity can be utilized as a new drug design strategy in the treatment of autoimmune diseases caused by excessive activation of the AIM2 inflammasome.

### P2X7-NLRP3 Inflammasome

The P2X7 receptor (P2X7R) is a ligand-gated nonselective cation channel, which serves as a unique member of the purinergic type 2 (P2) receptor family. The P2 receptor family consists of two main subfamilies: a P2X family of ligand-gated ion channel receptors (P2Y1, 2, 4, 6, 11−14) and a P2Y family of G protein-coupled receptors (P2X1–7) (54). The structure of the P2X7 monomer includes two α-helical transmembrane-spanning regions (TM1 and TM2) linked by a large extracellular loop containing ten conserved cysteine residues, which allow the formation of disulfide bonds, an intracellular N-terminal domain, and an obviously longer intracellular C-terminal domain compared to other P2X receptors (55). From the perspective of its three-dimensional structure, the shape of the P2X7 subunit resembles that of a dolphin (56).

The functional P2X7R, whose extracellular domains bind to three activator ATP molecules correspondingly, is composed of three intertwined P2X7 subunits. Among the P2X receptor family, P2X7R has the lowest affinity for ATP, such that a high ATP concentration is required for activation (57). The main source of ATP is pathological cell death, stress, plasma membrane rupture, and regulatory ATP release *via* pannexin-1, connexin-43, ATP-binding cassette (ABC), secretory vesicles, PRR activation, and P2X7R (58, 59). Stimulation of a low concentration of ATP will open the ATP-gated cation channel for a few milliseconds to facilitate the inflow of Na^+^ and Ca^2+^ and the outflow of K^+^. In contrast, a high concentration of ATP will trigger the formation of macropores across the plasma membrane within a few seconds to 1 min, which will permit the penetration of molecules with a molecular weight of up to 900 Da, such as Lucifer yellow, Yo-Pro, propidium, or ethidium (60).

Equally, the channel opening or pore formation caused by P2X7R activation has been identified as the main driving force of NLRP3 inflammasome activation. Particularly, P2X7R-mediated reduction of K^+^ in the cytoplasm promotes the interaction between the NLRP3 inflammasome and NEK7 (61). Besides, direct contact between P2X7R and NLRP3 inflammasomes at discrete subplasmalemmal cytoplasmic sites should also be considered. Franceschini et al. demonstrated that P2X7R and NLRP3 colocalize in mouse peritoneal macrophages and mouse microglia (62). Therefore, it is reasonable to list P2X7R as a separate chapter to emphasize not only its profound effect in NLRP3 inflammasome activation, but also its potential as a shortcut pathway in the occurrence of autoimmune diseases.

## Pathways in Pyroptosis

**Figure 1:**
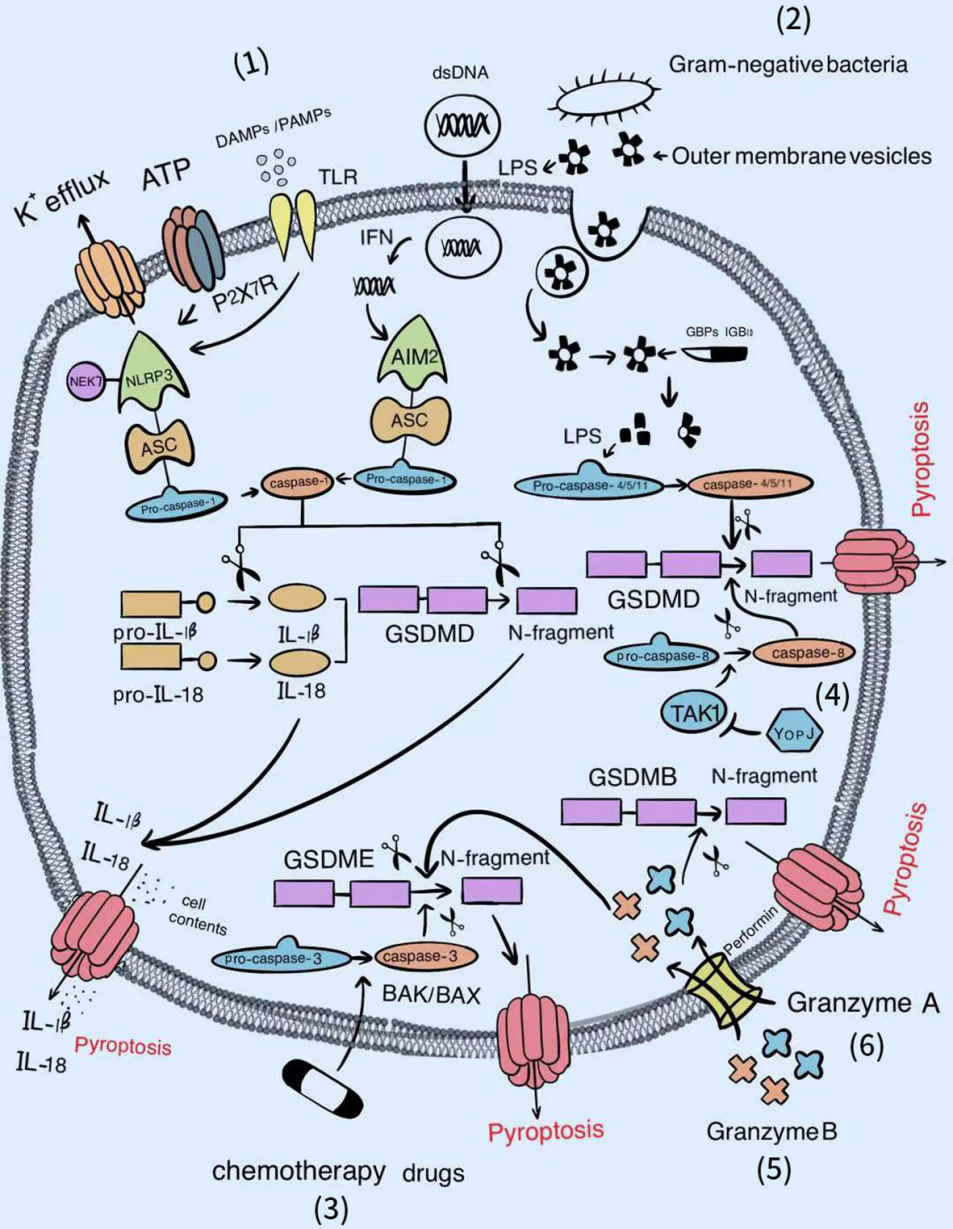
The signal pathways of pyroptosis. Various factors can activate the gasdermin famliy to trigger pyroptosis. (1) Classical inflammasomes/caspase-1/GSDMD-dependent pyroptotic pathway. (2) LPS/caspase-4, 5, or 11/GSDMD-dependent pyroptotic pathway. (3) Chemotherapy drugs/BAK/BAX/caspase-3/GSDME-dependent pyroptotic pathway. (4) YopJ/TAK1/caspase-8/GSDMD-dependent pyroptotic pathway. (5) Granzyme B/GSDME-dependent pyroptotic pathway. (6) Granzyme A/GSDMB-dependent pyroptotic pathway.

The dominant pathways of pyroptosis include the caspase-1 and caspase-4/5/11-dependent pathways. With the deepening of research, caspase-3-dependent and the caspase-free pathways have recently been reported. These new discoveries lay a solid foundation for expanding the territory of pyroptosis (​**Figure 1**).

### Caspase-1-Mediated Canonical Pathway

Once the classical inflammasome sensors (NLRs, AIM2, P2X7R, and pyrin) recognize pathogen-associated molecular patterns (PAMPs) or danger-associated molecular patterns (DAMPs), inflammasomes will assemble automatically (63). Subsequently, pro-caspase-1 proceeds self-cleavage to form the effective p10/p20 heterotetramer (64); this not only cleaves GSDMD to release the functional gasdermin N-terminal from the suppressive gasdermin C-terminal, but also cleaves the inactive precursor pro-IL-1β and pro-IL-18 into their respective mature secretory forms. Then, the gasdermin N-terminal migrates and adsorbs onto acidic lipids of the cell membrane, where it can generate negatively charged gasdermin pores 10–14-nm in inner diameter, promoting IL-1β and IL-18 discharge by electrostatic filtering and leading to cell burst and pyroptosis (65–67). In contrast, high mobility group box protein B1 (HMGB1) is an intracellular DAMP, and its release during pyroptosis was shown to be independent of gasdermin D pore but accompanied by cell lysis (68). Recent evidence suggested that plasma membrane rupture (PMR) after pore formation required the participation of Ninjurin-1 (NINJ1), a double transmembrane cell surface protein, rather than simply being a passive event. Reduced secretion of HMGB1 and cell retention of bubble morphology has been shown to occurred in NINJ1-deficient pyroptotic bone marrow-derived macrophages (BMDMs) (69). Therefore, NINJ1-related PMR can effectively enhance the host defense to microbial infections by releasing DAMPs to activate innate immunity while NINJ1 can also behave as a candidate target for suppressing excessive inflammation.

Benefiting from the activation of classical inflammasomes by various intracellular PAMPs, bacterial or viral DNA, and fungal hyphae (70), the induced pyroptotic cell death of infected cells can directly destroy the breeding grounds of pathogens, so as to minimize damage to the host. Thus, caspase-1-mediated pyroptosis represents a key defense pathway for the host in the context of extensive microbial infection. However, the excessive production of proinflammatory cytokines (mainly IL-1β and IL-18) in pyroptosis leads to a persistent of inflammatory state, resulting in inflammation and immune crosstalk. As pore formation serves as the final checkpoint for pyroptosis, drugs that block this critical step could offer considerable hope for therapies of pyroptosis-related autoimmune diseases. Either disulfiram or dimethyl fumarate (DMF) can modify Cys191/Cys192 in GSDMD of both human and mouse to diminish the capability of pore formation (71, 72). Regarding those already formed holes, the ESCRT-III complex, revealed by calcium influx, is devoted to restoring the plasma membrane for the sake of easing the privation of cell integrity (73). Furthermore, Kayagaki et al. found that IRF2 was essential for the transcriptional expression of the driller GSDMD. Moreover, IRF2 silencing significantly attenuated canonical and noncanonical inflammasome-mediated pyroptosis and IL-1β release (74), suggesting that IRF2 is a reliable drug target for the treatment of autoimmune disease.

### Caspase-4/5/11-Mediated Noncanonical Pathway

Dixit and Shao et al. successively ascertained that caspase-4/5 in humans, or caspase-11 in mice directly binds to lipid A of lipopolysaccharide (LPS) located at the outer membrane (OM) of Gram-negative bacteria with high specificity and affinity, inducing its own oligomerization and activation (75, 76). The driving force of this combination may be attributed partially the electrostatic attraction between the basic CARDs in caspase-4/5/11 and the acidic phosphate of the lipid A backbone in LPS. Later, active caspase-4/5/11 processes GSDMD to liberate the gasdermin N-terminal p30 fragment with pore-forming ability, followed by pyroptotic cell death (77, 78). The discovery that caspase-4 or caspase-5 in human and caspase-11 in mouse are capable of sensing intracellular LPS improves our previous understanding of the host surveillance of Gram-negative pathogens (79). Meanwhile, it perfectly compensates for the deficiency that only extracellular LPS can be detected by the Toll-like receptor 4 (TLR4)/myeloid differentiation-2 (MD-2) complex, thereby accomplishing the complete intracellular and extracellular clearance of LPS infection (80).

Nevertheless, the detailed mechanism underlying how lipid A from extracellular bacteria enters the cytoplasm is still being explored. A recent study demonstrated that once exposed to the inhabitable environment, Gram-negative bacteria increase the secretion of outer membrane vesicles (OMVs) whose main cargo is lipid A. Subsequently, OMVs are absorbed through clathrin-mediated endocytosis, ultimately unloading LPS at the cytosol from early endosomal compartments (81). Further research revealed that GBPs are first attracted by LPS to establish caspase activation platforms, and in turn facilitate the combination of caspase-4/11 and LPS with the assistance of interferon-inducible protein IRGB10 (82, 83). For bacteria that gain entry directly into the cell such as *F. novicida*, Man et al. indicated that after being recruited by GBPs, IRGB10 could co-localize with GBPs under the LPS layer encapsulation, collaborating to trigger the explosion of the bacterial outer membrane for adequate release of LPS (34). The remaining small part of free lipid A connected to HMGB1 released by hepatocytes, which were stimulated previously by circulating PAMPs such as LPS or poly (I:C), is then internalized into the lysosome of macrophages *via* the receptor for advanced glycation end products (RAGE). Subsequently, HMGB1 gradually permeabilizes the phospholipid bilayer under an acidic environment until the lysosomal membrane is destroyed, causing LPS to leak into the cytoplasm and activate caspase-11 (84). Moreover, secretoglobin 3A2 (SCGB3A2) secreted by epithelial cells of the respiratory airways, binds and promotes LPS access to the cytoplasm through interacting with the cell surface protein syndecan-1, thereby inducing pyroptosis (85). As cells from different parts of the body can take up LPS, it is no wonder that the noncanonical inflammasome activation by intracellular LPS can cause systemic clinical symptoms in autoimmune diseases.

Regarding the late stage of infection, pyroptotic innate immune cells express superfluous proinflammatory mediators and tissue factor (TF)-positive microvesicles, which is involved in the induction of blood coagulation and sepsis (86, 87). Consequently, it is essential to use inhibitors of the LPS-mediated noncanonical pyroptosis pathway to suppress hyperactive inflammation. Regarding promoter LPS, either glutathione peroxidase 4 (GPX4) attenuation of lipid peroxidation or heat shock protein A12A (HSPA12A)-mediated reduction of LPS in the cytoplasm has been shown to be an effective approach to restrain caspase-11-mediated pyroptosis (88, 89). Similarly, Serpin family B member 1 (SERPINB1) could restrict the CARD oligomerization of caspase-4/5/11, while Stearoyl lysophosphatidylcholine (LPC) prohibited caspase-11 from binding to LPS, both of which are negative regulators of caspase activation (90, 91). Even although more in-depth explorations of the noncanonical pyroptotic pathway in autoimmune diseases needed to be conducted, related inhibitors can be used in the first instance to prevent sepsis, which is particularly prevalent in patients with autoimmune diseases after receiving immunosuppressive therapy.

### Caspase-3-Mediated Emerging Pathway

In situations with a combination of high expression of GSDME with chemotherapy drug stimulation, caspase-3, generally classified as the apoptosis execution caspase, can cleave GSDME to obtain the gasdermin N-terminal that initiates pyroptosis (92). Subsequent studies further revealed that chemotherapy-induced pyroptosis mainly occurred through the BAK/BAX-caspase-3-GSDME pathway (93). Based on these discoveries, the strong adverse effects of chemotherapy drugs may be explained by the higher expression of GSDME in normal tissue cells compared to most cancer cells, which increases the ability of normal tissue cells to execute pyroptosis induced by chemotherapy, resulting in tissue damage and weight loss. On the contrary, in various tumors with high GSDME expression, chemotherapeutic drug-mediated pyroptosis is recognized as a powerful weapon to induce cancer cell death (94–96). Furthermore, we speculate that the exacerbation of the conditions of some patients with autoimmune diseases during the treatment process is closely related to the pyroptosis of normal cells caused by inappropriate medication, similar to the side effects caused by chemotherapeutic drugs, although further study is required to test this possibility.

Apart from caspase-3, caspase-8, as the upstream activator to regulate apoptotic cell death, has also been demonstrated to elicit pyroptosis by cleaving GSDMD in BMDMs infected with Yersinia. Specifically, Yersinia outer protein J (YopJ), the effector protein of the type III secretion system (T3SS) of pathogenic Yersinia, inhibited the activity of TGF-β activated kinase-1 (TAK1) to activate Receptor-Interacting Protein 1 (RIP1) and caspase-8 by virtue of its acetyltransferase activity (97, 98). Therefore, whether there are more caspases originally involved in apoptosis that can also mediate pyroptosis and under what conditions do these promote the transition of cell death type from apoptosis to pyroptosis require further exploration.

### Caspases-Free Pathway

Nevertheless, several recent studies have overturned the conventional belief that the gasdermin family can only be cleaved by the caspase family. Indeed, if the expression of GSDME in tumor cells was up-regulated accompanied by increasing tumor-infiltrating NK and CD8^+^ T lymphocytes, these killer cells could release perforin to form pores on their own cell membrane, permitting granzyme B to burst into the cytoplasm of target tumor cells, thereby cleaving GSDME after D270 to induce pyroptosis in a similar manner to caspase-3 (99). Furthermore, in patients with B cell leukemia, the occurrence of cytokine release syndrome (CRS) was associated with GSDME-mediated pyroptosis, which is also triggered by granzyme B liberated from chimeric antigen receptor (CAR) T cells (100). Remarkably, it has also been reported that granzyme A originating from cytotoxic lymphocytes supports cleaving GSDMB at Lys^244^ to debunk pore-forming fragments, and eventually encourages pyroptotic killing of GSDMB-expressing cells (101). Collectively, these findings indicate that the only reliable marker of pyroptosis seems to be the members of the gasdermin family.

## Role of Pyroptosis in Autoimmune Diseases

### Systemic Lupus Erythematosus (SLE)

The etiology of SLE such as environmental precipitants, hormonal factors, and genetic susceptibility can readily drive abnormal autoimmune reactions. As a result, immune complexes are extensively deposited in the kidneys, skin, blood vessels, brain and so forth, leading to impaired tissues and organs and establishing highly heterogeneous of clinical manifestations (102, 103). Recently, the relationship between pyroptosis and SLE has been gradually unraveled.

The latest evidence shows that the expression of NLRP3 inflammasome-related constituents expression were elevated in various cells, including bone marrow-derived mesenchymal stem cells and monocytes/macrophages, in patients with SLE, such as bone marrow-derived mesenchymal stem cells and monocytes/macrophages, and even the content of active caspase-1 in monocytes was positively correlated with the serum titer of anti-double stranded DNA antibodies (anti-dsDNA Abs) (104, 105). Anti-dsDNA Abs, the hallmark antibodies of SLE, can trigger NLRP3 inflammasome activation in monocytes/macrophages in patients with SLE by inducing mitochondrial ROS production and activating the TLR4-NF-κB signal pathway (106). Moreover, the latest analysis by Baxter et al. showed that monocytes that sustained pyroptosis after being treated with LPS/nigericin released numerous extracellular vesicles (EVs) (107), whose content, transport distance, and final destination remains a mystery worth further exploration. Nevertheless, as EVs can migrate freely to different organs of the body, they may be responsible for the pathogenic features of multisystem involvement in patients with SLE. Similarly, the highly expressed mammalian target of rapamycin (mTOR) in lupus mice has also been shown to induce lupus nephritis (LN) through activation of mTOR/mitochondrial ROS/NLRP3 signaling (108). Another function of anti-dsDNA Abs is to help DNA in the plasma or on the cell surface enter normal monocytes through endocytosis to activate the AIM2 inflammasome, forming a vicious cycle of SLE pathogenesis (109). Naik et al. recently revealed that epithelial stem cells (EpSCs) with stored inflammatory memories can accelerate wound tissue repair, in which pathway analysis showed that the AIM2 inflammasome and its downstream effectors IL-1β jointly participated (110). From another aspect, the memory of inflammation experience in EpSCs may lay the foundation for the frequent recurrence of autoimmune skin diseases such as SLE, where the AIM2 inflammasome may play the key role. M. Li et al. found that the content of P2X7R in Th17 cells in patients with SLE was evidently ascended and positively related the SLE Disease Activity Index (SLEDAI) score (111). However, Furini et al. pointed out that P2X7R expression on PBMCs was significantly reduced in patients with SLE (112). We hypothesize that the differential expression of P2X7R among different cell subtypes and the different disease duration of selected patients with SLE are the main reasons for the contradictory results. Therefore, to maximize the efficacy of P2X7R inhibitors in the treatment of SLE, it is necessary to continuously monitor the expression level of P2X7R in different disease courses and cells, and then administer P2X7R inhibitors in cases where P2X7R is highly expressed. Interestingly, researchers found that stimulation of bone marrow cells with bisphenol A (BPA), an environmental estrogen, could increase levels of NLRP3, while levels of Aim2 mRNA and protein also increased in cells treated with androgen (113, 114). Combined with the epidemiological findings that the incidence of SLE in women is much higher than that in men, it is reasonable to doubt that the NLRP3 inflammasome is more pathogenic than the AIM2 inflammasome to lead to this sex preference. The momentous duty of the NLRP3 inflammasome, AIM2 inflammasome, and P2X7 receptor, is as pyroptotic combustion improvers in the initiation and deterioration of SLE or even LN.

Strikingly, several new studies recently unveiled that, the AIM2 inflammasome, P2X7 receptor, and GSDMD play a double-sword function in the pathogenesis of SLE. Based on the study of Faliti et al., the P2X7 receptor participates in the occurrence of GSDMD-mediated pyroptosis in pathogenic T follicular helper (Tfh) cells, thereby preventing Tfh cells from assisting B cells in the germinal centers (GCs) to synthesize immunopathogenic IgG (115) and achieve immunopathology remission. Moreover, it is undeniable that AIM2 occupies a major position in the struggle to suppress IFN-β, a risk factor for SLE. As the Ube2i molecular chaperone, AIM2 can promote Ube2i-mediated sumoylation and inhibit the expression of IFN (116). Simultaneously, deficiency of the Aim2 gene was believed to increase the expression of IFN-inducible proteins such as STAT1 and p202. Indeed, p202 protein increased the production time of IFN-β by prolonging the half-life of AIM2 activator dsDNA, and also nourished IFN-β synthesis through the STING-TBK1-IRF3 pathway (117). Therefore, AIM2 protein is expected to become a novel target for reducing lupus susceptibility. Even GSDMD, regarded as the executioner of pyroptosis, has recently been found to assume part of the protective task in SLE. The mortality, pathogenic autoantibody synthesis, and inflammation in the kidney and lung of imiquimod­treated GSDMD^−/−^ mice were noticeably more intense than those of imiquimod­treated WT mice (118). One possible explanation for this phenomenon is that a lack of GSDMD leads to an increase in uncontrolled necrotic cell death locally, paving the way for autoantigen outflow and aggravation of autoimmune disturbance. These results suggest that we cannot consider the pathogenic effects of pyroptosis at only one point given that pyroptosis may also be the upstream source or downstream result of other immune or inflammatory responses; therefore, we should judge its overall effect from the whole inflammatory or immunologic cascade reaction.

It is well known that, except for the damage caused by pyroptotic cell death, cellular contents released from pyroptotic cells can also enhance immune-mediated inflammation in SLE. Evidence has shown that the nucleus of cells undergoing pyroptosis only demonstrate chromatin condensation, while the nucleus remains intact without nuclear rupture, which provides convenience for the production of crucial pathogenic factors antinuclear antibodies (ANA) in SLE (119, 120). In addition, IL-1β and IL-18 are released in large quantities, which could amplify the inflammatory response in the process of pyroptosis. Researchers have reported that compared to healthy controls (HCs), the serum IL-18 levels in patients with SLE were significantly increased and closely related to those in active LN, but there was no significant difference in the serum IL-1β level (121, 122). This may be because serum may be not the best source of samples for detecting IL-1β. However, there is no doubt that the attachment of IL-1βto the IL-1 receptor would activate the NF-κB pathway, accelerating the synthesis of downstream proinflammatory agents such as cyclooxygenase-2 (COX-2) and IFN-γ. The effect of IL-18 activating the p38-MAPK signaling pathway achieves an output increase in inflammatory cytokines, including IL-1α, IL-6, and IL-8 (123). Moreover, IL-1β and IL-18 can evoke surrounding neutrophils suffering NETosis to form a positive loop of inflammation in SLE (124, 125).

Furthermore, several studies have substantiated that the level of HMBG1 is increased in the serum, cutaneous lupus lesions, and urine and kidney biopsy samples of patients with SLE, among which, the level of serum HMBG1 was closely related to SLEDAI, and the level of urine HMBG1 depends on LN class (126–129). HMBG1 could not only aggrandize the expression of NF-κB-dependent pro-inflammatory factors in a TLR4-dependent manner, but can also combine with RAGE to induce pyroptosis of adjacent macrophages to expand the lesion area (130, 131). More importantly, the complex formed by HMGB1 with DNA, LPS, and histone has the potential to increase immunogenicity, worsening the autoimmune response (84, 132). The abundant excretion of the above inflammatory cell contents is the magic formula to trigger the chronicity and persistence of an inappropriate immune response in SLE.

To date, many drugs, such as baicalein, oleuropein, melatonin and piperine, have been illuminated to attenuate murine LN development by inhibiting NLRP3 inflammasome activation (133–136). Therefore, these specific inhibitors or bioactive substances, known to target the NLRP3 inflammasome, may be a novel strategy for treating not only kidney dysfunction but even other system involvement of SLE.

### Rheumatoid Arthritis (RA)

Synovial inflammation and progressive joint destruction are defining characteristics of RA (137). In RA, fibroblast-like synovial cells (FLS) and immune effector cells, such as monocytes/macrophages, and T cells secrete IL-1β and TNF-α, which have a series of pathological effects, including synovial cell proliferation, massive infiltration of inflammatory cells, pannus formation, and cartilage and bone tissue destruction (138). Several reports have demonstrated that pyroptosis participates in the process of RA.

According to the latest evidence, the synergistic effect of elevated pentaxin 3 (PTX3) and ligand C1q in the plasma of patients with RA activate the NLRP3 inflammasome in CD14^+^ monocytes to cause caspase-1-mediated pyroptosis and inflammatory cytokines (IL-1β, IL-18, IL-6, and TNF-α) excretion, the degree of which was consistent with disease activity. In turn, IL-6 emission facilitates PTX3 plus C1q-induced monocyte pyroptosis (139), thus forming a positive inflammatory feedback of RA. In addition, IL-6 with ATP assistance, utilized the cathepsin B/S100A9 pathway to activate the NLRP3 inflammasome, promoting collagen-induced arthritis in mice (140). Moreover, the dual signaling of TNF-α and calreticulin has been proven to activate the NLRP3 inflammasome and increase IL-1β expression in FLS (141), the main outcome of which is the transformation of the synovial membrane into proliferative invasive tissue to destroy the cartilage and bone.

Recently, accumulating studies have demonstrated that extracellular acidosis can also erode the articular cartilage in RA *via* increasing secretion of IL-1β (142, 143), which is closely pertained to pyroptosis. Wu et al. reported that acid-sensitive ion channel 1a (ASIC1a) up-regulated the contents of the NLRP3 inflammasome and IL-1β by increasing the influx of Ca^2+^ into cells, thereby triggering articular chondrocytes pyroptosis (144). The latest research indicated that the calpain-2/calcineurin pathway downstream of ASIC1a may contribute to acid-induced pyroptosis of articular chondrocytes (145). Moreover, low expression of circular RNA Hsa_circ_0044235 in patients with RA was corroborated to rely on the miR-135b-5P-SIRT1 axis to expedite the occurrence of NLRP3-mediated pyroptosis of chondrocytes (146). Therefore, the occurrence of pyroptosis on chondrocytes may be the primary cause of the articular cartilage defects in patients with RA, and it seems fair to suspect that destruction of bone tissue in the late stage of RA is also related to pyroptosis. Another study showed that lower expression of the DNA nuclease MRE11A in the CD4^+^T cells of patients with RA compared to HCs caused mtDNA oxidation and leakage into the cytosol, inducing activation of the NLRP3 and AIM2 inflammasomes to guide CD4^+^T cell pyroptosis (147). This is a momentous discovery that urges CD4^+^T cells to join the ranks of chronic inflammatory cells and greatly enriches the source of inflammatory mediators in adaptive immune responses.

Regarding P2X7R, the expression of P2X7 mRNA in patients with RA has been shown to be significantly up-regulated compared to the HCs (148). Further studies by Dong et al. found that anticitrullinated protein antibodies (ACPAs), as RA-specific autoantibodies, could activate pannexin channels to induce release of ATP, resulting in P2X7-NLRP3 inflammasome activation in macrophages and the maturation of IL-1β (149). Consequently, ACPA seropositivity can be used at an early stage as an independent risk factor to predict the probability of joint injury and disability in patients with RA, and ACPA may also serve as a promising drug target for inhibiting IL-1β production. Meanwhile, it has been confirmed that increased extracellular Ca^2+^ concentrations ([Ca^2+^]_ex_) derived from local bone erosion or dying cells in the joints contribute to the formation of calciprotein particles (CPPs). Then, monocytes absorb CPPs through calcium-sensing receptor (CaSR) in response to increased [Ca^2+^]_ex_-stimulated macropinocytosis, which activate the NLRP3 inflammasome, leading to the release of IL-1β and increased cell death (150). Although it has not been determined whether pyroptosis is involved in monocyte death, it is undeniable that monocytes are the main source of the cartilage degradation mediator IL-1β in RA.

Intriguingly, according to recent studies, in addition to traditional IL-1β inhibitors, NLRP3 or P2X7R blockers also reduce the release of cartilage destruction factor IL-1β, and alleviate joint inflammation by controlling macrophage or FLS pyroptosis (151–153), which increases the potential therapeutic targets of RA.

### Inflammatory Bowel Disease (IBD)

As a common chronic gastrointestinal autoimmune disease, IBD mainly includes two subtypes of ulcerative colitis (UC) and Crohn’s disease (CD), representing inflammatory disorders confined to the colon or affecting the entire gastrointestinal tract, respectively. The pathogenesis of IBD touches upon intestinal microbiota disorder, immune system homeostasis imbalance, environmental factors and genetic susceptibility (154–156). Numerous studies have called attention to the close interaction between pyroptosis and IBD.

Firstly, X. Chen et al. found that either the mRNA expression or protein levels of NEK7 and other pyroptosis-related components, including NLRP3, caspase-1, and GSDMD, in UC tissues were significantly higher than those in control tissues. Further, knocking out NEK7 was shown to eliminate ATP+LPS-induced intestinal epithelial MODE-K cell pyroptosis or reduce the symptoms of dextran sulfate sodium (DSS)-induced colitis in mice (157). These findings suggest that the contribution of NEK7 to NLRP3-mediated IEC pyroptosis should not be underestimated, and that blocking NEK7 may become a welcome sign in IBD treatment. Similarly, CD147, also named basigin, has been confirmed to trigger IEC pyroptosis in IBD by activating the NF-κB pathway, causing massive IL-1β and IL-18 discharge (158). Subsequently, IL-1β disrupts intestinal epithelial tight junctions, resulting in increased permeability of intestinal epithelium, and also coordinates with IL-6 to induce the differentiation of naive T cells into Th17 cells, which maintain the inflammatory state (159, 160). IL-18 destroys the intestinal mucosal barrier through aggravating loss of mature goblet cells, resulting in microflora-driven intestinal inflammation (161). Deeper studies have claimed high expression of monocarboxylate transporter 4 (MCT4) exacerbates intestinal inflammation in patients with IBD *via* activation of the NLRP3 inflammasome through the ERK1/2-NF-κB axis, which initiates pyroptosis in IECs (162, 163). Moreover, numerous microRNAs (miRNAs)-related active agents have been shown to ameliorate colitis by abrogating cell pyroptosis, namely human umbilical cord mesenchymal stem cell (hucMSC)-derived exosomal miR-378a-5p, which inhibits NLRP3 inflammasome assembly (164), and *Roseburia intestinalis*-derived flagellin *via* blocking the miR-223-3p/NLRP3 axis to decreaseNLRP3 inflammasome activation (165).

As confirmed recently, the extracellular ATP level in the colon tissue of DSS-induced colitis mice was significantly enhanced as compared to control; however, the symptoms were markedly improved by injection of apyrase, an ATP diphosphohydrolase, or P2X7R inhibitor A438079 (166). *In vitro*, Diezmos et al. successfully applied a pannexin-1 channel blocker or A438079 to control typical IBD lesions in colonic mucosal strips, such as crypt injury, loss of tight junction, and increase in cell permeability (167). Indeed, P2X7R oral inhibitors have already entered the clinic. AZD9056 has been shown to be effective in lowering the CD Activity Index (CDAI) and relieving chronic abdominal pain in adult patients with moderately to severely active CD (168). Additionally, the activation of P2X7 receptors during colitis could also mediate the death of enteric neurons, causing colonic motor dysfunction, or induce mucosal Treg cell death, leading to aggravation of inflammation (169–172). Although these studies indicate the pathogenicity of P2X7R preliminarily in IBD, whether P2X7R-mediated pyroptosis is associated with the abovementioned clinical symptoms of IBD requires stronger evidence. According to the latest reports, the swelling and shedding of IECs fosters an intimate relationship with the pyroptosis caused by the TNF-α/IRF1/caspase-3/GSDME pathway (173). The catastrophic outcome related to this is the flow of HMGB1 from pyroptotic IECs, which contributes to the proliferation of cancer cells in colitis-associated colorectal cancer *via* the ERK1/2 pathway (174). Consequently, inhibition of IEC pyroptosis may be a new approach for the early prevention or treatment of colitis-related tumors.

Several recent studies have announced that the number of cells per 1000 IECs that undergo pyroptosis was associated with clinical response and endoscopic improvement of patients with CD to vedolizumab (175). Indeed, the serum levels of NLRP3 and HMGB1 have been found to be positively correlated with the severity of UC (176). Therefore, it is noteworthy that the incidence of pyroptosis in IECs and the serological levels of pyroptosis-related components proteins may perform well in predicting prognosis as well as noninvasive assessment of disease activity for patients with IBD.

### Sjogren’s Syndrome (SS)

The main symptoms of SS are xerophthalmia and xerostomia, which are and are often accompanied by skin, bone, kidney, lung damage, lymphoma and other system manifestations. The histopathological feature of SS is the progressive infiltration of lymphocytes into the exocrine glands, which produce inflammatory agents to accentuate the degeneration of exocrine glands (177–179). Until fairly recently, some laboratories have provided evidence of pyroptosis in the exocrine glands of patients with SS.

Current studies confirmed that compared to HC, the expression of NLRP3 inflammasome-related elements in PBMC or macrophages infiltrating into the salivary glands of patients with SS increased (180, 181). As for NLRP3 inflammasome-mediated pyroptosis, the culprit was massive inflammatory circulating cell-free DNA (cf-DNA) accumulated in the serum, the cytoplasmic part of PBMCs, and the salivary gland tissue of patients with SS (181). The two major factors that promoted inflammatory DNA deposition were significantly reduced activity and expression of DNase, which led to blood-derived cf-DNA supersaturation, as well as necrotic chromatin release from the pyroptotic macrophages infiltrating salivary glands, thus forming an inflammatory vicious cycle. Similarly, due to selective DNase1 deficiency, the exorbitant accumulation of damaged cytoplasmic DNA in the ductal salivary epithelia of patients with SS can activate the AIM2 inflammasome, causing intensive expression of pyroptosomes in the same region (182). Moreover, type I IFN up-regulated the expression of caspase-1 and GSDMD in salivary gland epithelial cells (SGECs) of patients with SS and may accelerate NLRP3 or AMI2 inflammasome-associated pyroptosis (183). Investigators have also found that after injecting P2X7R antagonist A438079 into a mouse model of salivary gland inflammation, there was an evident advance in saliva flow accompanied by a decrease in lymphocyte infiltration in the submandibular gland. This finding implies that the P2X7R/NLRP3 inflammasome/caspase-1/IL-1β and IL-18 axis may partake in the pathogenesis of SS (184, 185). Taken together, these results clearly indicate that the number of SGECs will decrease due SGEC pyroptosis, thereby resulting a considerable drop in the amount of saliva secretion. More seriously, the inflammatory cytokines (IL-1β/IL-18) excreted by the pyroptotic SGECs may cause the infiltration and activation of immune cells in the salivary glands and induce the dysfunction of adjacent normal SGECs.

Notably, P2X7R-NLRP3 inflammasome complex expression levels in the salivary glands of patients with SS were positively correlated with the incidence of mucosa-associated lymphoid tissue non-Hodgkin’s lymphoma, although the pyroptosis-specific mechanism in this phenomenon is still under investigation (186). Furthermore, there is still a lack of reports on lacrimal gland epithelial cells pyroptosis, which even decreases tear secretion in patients with SS; however, pyroptosis-related inhibitors may replace artificial tears to guide a new treatment direction for dry eyes to prevent lacrimal gland epithelial cell loss.

### Dermatomyositis (DM)

DM is a rare idiopathic inflammatory myopathy. The lesions mainly involve the skin and muscles, and are often accompanied by systemic complaints such as pulmonary interstitial lesions, dysphagia, tumors, and diastolic dysfunction (187). Muscle biopsy specimens of patients with DM are histopathologically characterized by perifascicular atrophy (PFA) and inflammatory cell infiltration (188). Recent evidence has shown that cell pyroptosis contributes to the pathological process of PFA in DM.

Preliminary studies have shown marked elevation of serum IL-1β and IL-18 levels, as well as the protein expression of NLRP3 and caspase-1 in muscle samples in patients with DM (189). D. Liu et al. further demonstrated that pyruvate kinase isozyme M2 (PKM2), not only the main rate-limiting enzyme of glycolysis but also the activation signal of NLRP3 inflammasome, was highly expressed in the muscle tissues of patients with DM, subsequently facilitating GSDMD-mediated pyroptosis of skeletal muscle cells (190). Another possible explanation for PFA is that overexpression of GSDME in the muscle fibers can convert mitochondrial apoptosis into mitochondrial pyroptosis. BAX/BAK located in the mitochondrion increases the permeability of the mitochondrial outer membrane to prepare for the release of cytochrome C, leading to the occurrence of cytochrome C/caspase-9/caspase-3/GSDME-mediated pyroptosis in myofibers (191). In addition, the latest study by Chai et al. found that the caspase-11-mediated noncanonical pathway of pyroptosis was involved in the pathogenesis of experimental autoimmune myositis in mice (192). However, further investigation is needed to understand whether the corresponding caspase-4/5-mediated noncanonical pyroptosis pathway exists in patients with DM. Although the pyroptosis of skeletal muscle cells seems to be the reasonable explanation for PFA in patients with DM, it is of concern that the muscle symptoms of patients with DM are mainly symmetrical proximal muscle weakness (193). Therefore, whether there is a preference of the limb location for muscle cells going through pyroptosis and the mechanism behind this uneven distribution still warrants further examination. It is noteworthy that glucocorticoids have recently been confirmed to induce skeletal muscle atrophy through the NLRP3/caspase-1/GSDMD pathway (194, 195), and glucocorticoids are the first choice in the treatment of patients with DM. Therefore, there may be pathological and pharmacological crosstalk in the occurrence of PFA in the later stage of DM, leading to the use of glucocorticoids should be more cautious.

Additionally, emerging evidence has manifested that the increased IL-18 in the skin lesions of patients with DM is mainly released by keratinocytes (196), epidermal atrophy occurs in patients with DM (193), and sun exposure is an environmental risk factor of DM flare (197). Meanwhile, several studies have found that Ultraviolet B (UVB) can trigger keratinocytes pyroptosis (198, 199). Consequently, whether the UVB and other environmental factors induced keratinocytes pyroptosis and release of inflammatory cytokines (IL-18 and IL-1β) can be responsible for the skin histopathological features and even systemic manifestations of DM undoubtedly deserve further studies.

## Conclusions and Perspectives

Recently, research on both pyroptosis and autoimmune diseases has progressed rapidly. An accumulating body of evidence has affirmed that pyroptosis acts an indispensable part in pathogenesis of autoimmune diseases, such as pyroptotic microglia and oligodendrocytes in multiple sclerosis (MS), pyroptotic thyroid follicular cells in Hashimoto’s thyroiditis (HT), and pyroptosis-related gene variants in pemphigus foliaceus (PF) (200–202). In this review, we depict known pyroptosis pathways in different cells and tissues of well-studied autoimmune diseases (**Table 1**), and describe the incidence characteristics and clinical manifestations related to pyroptosis. However, there are still many puzzles to be solved and areas to be explored.

**Table 1:** Summary of pathway and role of pyroptosis in autoimmune diseases.

Diseases	Activators	Inflammasomes	Caspase family	Gasdermin family	Pyroptotic cells	Function	References
SLE	LPS and ATP	NLRP3	Caspase-1	GSDMD	Proximal tubular epithelial HK-2 cells	Lupus nephritis	(136)
	eATP	P2X7-NLRP3	Caspase-1	GSDMD	T follicular helper cells	Reduce the synthesis of autoimmune antibodies	(115)
RA	C1q and PTX3	NLRP3	Caspase-1	GSDMD	CD14^+^ monocytes	Aggravate inflammation	(139)
	Extracellular acidosis	NLRP3	Caspase-1	GSDMD	Chondrocytes	Cartilage destruction	(144, 146)
	Hypoxia	NLRP3	Caspase-1	GSDMD	FLS	Aggravate inflammation	(151)
	mtDNA	NLRP3	Caspase-1	GSDMD	CD4^+^ T cells	Aggravate inflammation	(147)
	mtDNA	AIM2	Caspase-1	GSDMD			
IBD	LPS and ATP MCT4	NLRP3	Caspase-1	GSDMD	Intestinal epithelial cells	Intestinal barrier impairment	(157, 163);
	CD147 CD147	NLRP3	Caspase-1	GSDMD			(158)
				GSDME			
	IRF1		Caspase-3	GSDME			(173)
SS	cf-DNA	NLRP3	Caspase-1	GSDMD	Macrophages infiltrating in the salivary gland	Loss of saliva secretion	(181)
	cytoplasmic DNA	AIM2	Caspase-1	GSDMD	Salivary gland epithelial cells		(182, 183)
DM	PKM2-dependent glycolysis	NLRP3	Caspase-1	GSDMD	Skeletal muscle cells	Perifascicular atrophy	(190)
	Mitochondrial damage		Caspase-3	GSDME	Myofibers		(191)

eATP, extracellular ATP; C1q, complement C1q; PTX3, pentaxin 3; FLS, fibroblast-like synovial cells; mtDNA, mitochondrial DNA; MCT4, monocarboxylate transporter 4; CD147 also known as Basigin; IRF1, interferon regulatory factor1; cf-DNA, circulating cell-free DNA; PKM2, pyruvate kinase isozyme M2.

Due to the diversity of the inflammasomes, the caspase family, and the gasdermin family in the pyroptosis pathways, there are clear differences in the combination of these participants between different autoimmune diseases. Even in the same disease, pyroptosis may occur in different effector cells, and there are multiple pyroptosis pathways in the same cell. Therefore, it is necessary to comprehensively evaluate whether pyroptosis plays a pathogenic or protective role in certain diseases, which is also applicable to the evaluation of the role of pyroptosis in tumor progression. Pyroptosis, as an inflammatory programmed cell death, directly prevents tumor proliferation and metastasis after the death of cancer cells. Moreover, pyroptotic cancer cells release a variety of DAMPs to activate immune cells, constructing the anti-tumor immune microenvironment. Released inflammatory cytokines may also induce cell carcinogenesis through chronic inflammation. Thus, whether pyroptosis plays an anti-tumor or a tumor-promoting role depends on the type of tumor, the decisive pyroptosis pathway, and the expression levels of pyroptosis pathway-related proteins in cancer cells. Furthermore, the current identification of the occurrence of pyroptosis is mainly through scanning electron microscopy, to observe cell morphology or q-PCR/western blot to detect the expression level of classical pyroptosis-related genes or proteins; some laboratories tend to choose the latter for verification due to the limitation of technical conditions. Nevertheless, in the newly discovered pathway of pyroptosis, molecules previously considered to be “classical” may not be necessary, and many novel molecules have emerged at the same time. Therefore, if it is not combined with morphological analysis, it is likely to draw a false negative conclusion. It is expected that more sensitive, more specific, and more convenient markers can be exploited in the future to evaluate the severity of pyroptosis, which may be in accordance with the therapeutic effect and prognosis of the autoimmune diseases. Additionally, various inhibitors targeting small molecules that play key roles in pyroptosis pathways have shown efficacy in clinical trials, showing broad application prospects.

In conclusion, efforts should be taken to further consummate the complete signaling pathway and underlying role of pyroptosis in more autoimmune diseases, with the aim to usher in a new era for treating autoimmune diseases.

## Author Contributions

RY: drafted the manuscript, drew the figures and summarized the table. XH, ZZ, and YZ: collected literature and prepared the related literature. YX: discussed and revised the manuscript. RX: designed the study, reviewed and edited the paper. All authors have read and approved the final manuscript.

## Funding

This work was supported by the National Natural Science Foundation of China (NO.81371744, 81773333, 82073449, and 82003363).

## Conflict of Interest

The authors declare that the research was conducted in the absence of any commercial or financial relationships that could be construed as a potential conflict of interest.

## Publisher’s Note

All claims expressed in this article are solely those of the authors and do not necessarily represent those of their affiliated organizations, or those of the publisher, the editors and the reviewers. Any product that may be evaluated in this article, or claim that may be made by its manufacturer, is not guaranteed or endorsed by the publisher.

## Glossary

ASC: apoptosis-associated speck-like protein containing a CARD
NLRs: nucleotide-binding leucine-rich repeat proteins
LRR: leucine-rich repeat
ATPase: adenosine triphosphatase
PYD: pyrin domain
CARD: caspase activation and recruitment domain
TLR: Toll-like receptor
IL-1R: leukin-1 receptor
TNFR: tumor necrosis factor receptor
MTOC: microtubule-organizing center
ROS: reactive oxygen species
NEK7: NIMA-related kinase 7
HDAC6: histone deacetylase 6
HD2: helical domain 2
PTM: post-translational modification
AIM2: absent in melanoma 2
PYHIN: pyrin and HIN domain-containing
EV71: enterovirus 71
mtDNA: mitochondrial DNA
type I IFN: Type I interferon
IFNAR: type I IFN receptor
IRF1: interferon regulatory factor 1
GBPs: guanylate binding proteins
TRIM11: tripartite motif 11
P2X7R: P2X7 receptor
P2: purinergic type 2
ABC: ATPbinding cassette
PAMPs: pathogen-associated molecular patterns
DAMPs: danger-associated molecular patterns
HMGB1: high mobility group box 1
PMR: plasma membrane rupture
NINJ1: Ninjurin-1
BMDMs: bone marrow-derived macrophages
DMF: dimethyl fumarate
LPS: lipopolysaccharide
OM: outer membrane
MD-2: myeloid differentiation-2
OMVs: outer membrane vesicles
RAGE: receptor for advanced glycation end products
SCGB3A2: secretoglobin 3A2
TF: tissue factor
GPX4: glutathione peroxidase 4 1
HSPA12A: heat shockprotein A12A
SERPINB1: Serpin family B member 1
LPC: lysophosphatidylcholine
YopJ: Yersinia outer protein J
T3SS: type III secretionsystem
TAK1: TGF-β activated kinase-1
RIP1: Receptor-Interacting Protein 1
CRS: cytokine release syndrome
CAR: chimeric antigen receptor
SLE: systemic lupus erythematosus
anti-dsDNA Abs: anti-double stranded DNA antibodies
EVs: extracellular vesicles
mTOR: mammalian target of rapamycin
LN: lupus nephritis
EpSCs: epithelial stem cells
SLEDAI: SLE Disease Activity Index
BPA: bisphenol A
Tfh: T follicular helper
GCs: germinal centers
ANA: antinuclear antibodies
HCs: healthy controls
COX-2: cyclooxygenase-2
RA: rheumatoid arthritis
FLS: fibroblast-like synovial cells
PTX3: pentaxin 3
ASIC1a: acid-sensitive ion channel 1a
ACPAs: anticitrullinated protein antibodies
[Ca^2+^]ex: extracellular Ca^2+^ concentration
CPPs: calciprotein particles
CaSR: calcium-sensing receptor
IBD: inflammatory bowel disease
UC: ulcerative colitis
CD: Crohn’s disease
DSS: dextran sulfate sodium
MCT4: monocarboxylate transporter 4
miRNAs: microRNAs
hucMSC: human umbilical cord mesenchymal stem cell
CDAI: CD Activity Index
SS: Sjogren’s syndrome
cf-DNA: circulating cell-free DNA
SGECs: salivary gland epithelial cells
DM: dermatomyositis
PFA: perifascicular atrophy
PKM2: pyruvate kinase isozymeM2
UVB: Ultraviolet B
MS: multiple sclerosis
HT: Hashimoto’sthyroiditis
PF: pemphigus foliaceus
